# Progress in brain barriers and brain fluid research in 2017

**DOI:** 10.1186/s12987-018-0091-8

**Published:** 2018-02-02

**Authors:** Richard F. Keep, Hazel C. Jones, Lester R. Drewes

**Affiliations:** 1 Department of Neurosurgery, University of Michigan, R5018 BSRB, 109 Zina Pitcher Place, Ann Arbor, MI 48105 USA; 2 Gagle Brook House, Chesterton, Bicester, OX26 1UF UK; 3 Department of Biomedical Sciences, University of Minnesota Medical School Duluth, Duluth, MN 55812 USA

## Abstract

The past year, 2017, has seen many important papers published in the fields covered by *Fluids and Barriers of the CNS*. This article from the Editors highlights some.

## Editorial

The purpose of this editorial is to highlight advances in brain barriers and brain fluids research in 2017 as well as areas of debate. As always, it is not possible to cover all important progress and, as we have mentioned before [1], such choices are idiosyncratic. However, we hope this editorial is useful for informing our readership and identifying promising areas for study as well as areas where technological advances are needed.

## Blood–brain barrier (BBB)/neurovascular unit (NVU)

There continues to be progress in identifying pathways important for the development of NVU/BBB properties. Thus, Cho et al. found [2] that the endothelial G-protein-coupled receptor (GPCR) Gpr124 and a glycosylphosphatidylinositol-anchored membrane protein, Reck, are required for forebrain angiogenesis and acquisition of brain barrier properties in mouse development. Both molecules appear to function by regulating Wnt signaling. Chang et al. [3] further examined the effect of a conditional knockout of Gpr124 in adult mice. No BBB effect was observed under normal conditions, but increased barrier disruption occurred in ischemic stroke and glioblastoma models. The effects of Gpr124 were again via the Wnt-βcatenin pathway, with activation of Wnt-βcatenin signaling reversing the effect of the Gpr124 conditional knockout.

Pericytes continued to be a major focus of current NVU/BBB research. Nakazato et al. [4] reported that a circadian clock transcriptional activator, brain and muscle aryl hydrocarbon receptor nuclear translocator-like protein 1 (Bmal1), is an important regulator of pericyte function. Bmal1 deletion caused pericyte dysfunction, age-dependent loss of pericytes, and endothelium hyperpermeability. The possible role of pericytes in circadian changes in barrier function merits further investigation. Some of the evidence for the importance of pericytes in NVU/BBB regulation has come from mice with a mutation in the retention motif for platelet derived growth factor (pdgf)-β (pdgf-b^ret/ret^) which have pericyte depletion. A recent study found that the effects of such depletion on NVU/BBB permeability were brain region-dependent (e.g. greater in cortex, striatum and hippocampus) [5]. There has been interest in whether NVU/BBB function differs between brain regions and these results suggest that there are differences in regulation. Although most studies on pericytes described beneficial effects of pericytes on BBB function, they may have potentially detrimental effects. Underly et al. [6] found that pericytes caused early BBB damage after cerebral ischemia via a matrix metalloproteinase9-dependent mechanism.

A potential theme is developing on the importance of lipid regulation in NVU/BBB function. More et al. found that peroxisome proliferator-activated receptor (PPAR) α is not only a lipid sensor, but it also regulates the expression and activity of brain endothelial efflux transporters [7]. In addition, Andreone et al. found that lipid transport by Mfsd2a inhibits caveolae vesicle formation in brain endothelial cells suppressing transcytosis [8]. These results suggested that the Mfsd2a gene product may have a dual role in lipid metabolism/transport and transcytosis. Similarly, recent evidence suggested the importance of alterations in sphingosine-1-phosphate signaling in BBB dysfunction after endotoxemia [9].

As with other tissues, there was great interest in the role of microRNAs and exosomes in brain and cells of the NVU. Thus, for example, Xi et al. [10] found that microRNA-126-3p promotes barrier integrity in the setting of intracerebral hemorrhage. The therapeutic use of using microRNAs to treat neurological conditions is potentially a beneficial area of research, but delivery into the endothelial cells and brain parenchyma remains a major issue [11]. The use of exosomes as a delivery system is one approach being vigorously pursued [12, 13]. For both exosomes and/or microRNA, the cerebral endothelium may be an easier therapeutic target than the brain parenchyma.

## Modifying the BBB for drug delivery

Currently, different approaches are being tested to modify brain endothelial tight junctions and thereby enhance drug delivery to the brain. Hashimoto et al. have shown that a monoclonal antibody targeting claudin-5 can increase barrier permeability in an in vitro endothelial barrier model [14]. Similarly, Dithmer et al. showed that peptidomimetics that have nanomolar affinity for claudin-5 increase barrier permeability in vitro and in vivo [15], a feature that was reversible in 12–48 h. This is important considering the potential side-effects of modifying claudin-5 [16] (see below).

Clinically, osmotic-induced blood–brain disruption is currently used to enhance delivery of anti-neoplastic agents to brain tumors [17]. An important consideration is the potential effect of the anti-neoplastic agent on normal tissue. Dosa et al. [18] reported the results of an early stage clinical trial of *N*-acetyl cysteine to reduce the ototoxic side-effects induced by cisplatin.

A number of different approaches were investigated to improve drug transfer to the brain: peptide vectors including antibodies may be used to target the LDL receptor and transfer ligands by receptor-mediated transcytosis [19, 20]. Shimizu et al. [21] observed that an antibody against an endothelial membrane protein (glucose-regulated protein 78) led to tight junction disruption and enhanced permeability to high molecular weight proteins. Thus, development of a strategy for controlled delivery of biologics, such as proteins or genes, to the brain may be possible. Also, specific gene therapy with adeno-associated viral vectors was tested in mice to control seizures [22]. Another approach to focally enhance brain vascular permeability was the use of ultrasound and microbubbles [23], currently in clinical investigation for Alzheimer’s disease therapy: Blood Brain Barrier Opening in Alzheimer’ Disease trial (BOREAL1; NCT03119961).

## Barriers in disease

Many neurological conditions (e.g. ischemic and hemorrhagic stroke, multiple sclerosis and neurodegenerative diseases) impact the NVU/BBB [24, 25] and blood-CSF barriers [26, 27]. Recently, Menard et al. [16] extended such findings by examining the effects of social stress in mice (a model of depression). They found that stress induced by chronic social defeat reduced brain microvessel claudin-5 expression in the nucleus accumbens, and that reducing claudin-5 with a short hairpin RNA caused depression-like symptoms and increased entry of interleukin-6 into brain. Interestingly, the effects on claudin-5 were reversed by antidepressant treatment. Other studies have indicated that there are subtle NVU/BBB changes in a variety of conditions including aging [24] and cerebral small vessel disease [28]. The impact of these changes (e.g. low level neuroinflammation) is an important area for investigation.

Over the decades there has been a longstanding debate over the relative importance of alterations in the paracellular and transcellular pathways in disease-induced modification of blood–brain transport. Currently, there is a debate about the relative importance of tight junction modification vs. transcytosis. It should be noted that there may be important interactions between tight junctions and the vesicular system (e.g. in internalization of tight junction proteins from the plasma membrane [29]) complicating data interpretation and that there may be differing results dependent upon which markers are being used to assess barrier function. In ischemic stroke, the importance of changes in transcytosis [30] and tight junctions [31] was recently highlighted. An issue with regards to changes in NVU/BBB function in neurological conditions is whether it is a consequence of the condition or whether it contributes to the injury. It is important, therefore, that Shi et al. [32] found that ameliorating BBB disruption in ischemia/reperfusion injury in mice by overexpressing heat shock protein-27 specifically in the endothelium, reduced overall stroke-induced brain injury (infarct size and neurological deficits). Such results indicate that the BBB is a therapeutic target for stroke.

The ultimate goal of brain barrier and brain fluid research is to improve patient outcome. In this regard, the potential use of glibenclamide (glyburide) to reduce brain edema for a variety of neurological conditions is noteworthy. Glibenclamide is a Sur1-TRPM4 channel inhibitor that has been shown to reduce brain edema in a variety of preclinical models (e.g. [33, 34]). It is in clinical trial for stroke-induced brain edema (NCT02864953) and to reduce edema in metastatic brain tumor patients receiving radiosurgery (NCT02460874).

## Choroid plexus, CSF secretion and CSF outflow

There has been some debate of the relative role of the choroid plexus in CSF secretion [1]. Praetorius and Dimiker [35] produced a comprehensive review of vectorial ion transport at the choroid plexus epithelium forming the basis for fluid secretion. Such ion transport is not only important for fluid secretion it is also involved in CSF ion homeostasis and secondary active transport. One focus of that review was Na, K and Cl transport by the choroid plexus epithelium. Interestingly, recent evidence indicated that stimulation of choroid plexus Na–K–Cl cotransporter-1 (NKCC-1; Slc12a2) contributed to post-hemorrhagic hydrocephalus [26] (see below). There was also growing evidence for the role of the choroid plexus in neuroinflammation. Thus, results indicated that the choroid plexus is a key site for the entry of T cells into brain in both animal and human stroke [36]. A potentially interesting model for studying choroid plexus development and function was described by Koshida et al. [37]. They found that mice with the transcription factor MafB gene knocked out had delayed differentiation and hypoplasia of the hindbrain choroid plexus, along with increased apoptosis and reduced proliferation in the epithelium.

There has long been substantial data that much CSF absorption is not via the arachnoid granulations/villi into the blood stream but rather into the lymph system via multiple routes [38]. The latter includes CSF drainage through the cribriform plate to the nasal lymphatics and the cervical lymph nodes and drainage via the spinal nerve roots to the lumbar lymph nodes [38], as well as via lymph vessels within the dura [39, 40]. Recently, Ma et al. [41] used noninvasive imaging techniques to quantify the transport of different sized tracers from CSF to the lymph nodes or blood in mice. For that species, they found that the lymph route predominated for both large and small tracers and that such drainage decreased with age.

## Fluid and solute flow within the brain

The proposed brain glymphatic system for the brain continued to generate much interest [42], with ~ 80 papers in PubMed in 2017. It is proposed that fluid flow within the brain occurs via the perivascular space around the arterial system, then through astrocytes, with water movement via aquaporin-4, leaving the brain via the perivascular space around the venous system. Altered flow was proposed to occur and contribute to a multitude of neurological conditions (e.g. Alzheimer’s disease, idiopathic normal pressure hydrocephalus, migraine, diabetes, traumatic brain injury and stroke [43–48]). Burfeind et al. examined whether five aquaporin-4 single-nucleotide polymorphisms (SNPs) were associated with Alzheimer’s pathology or rate of cognitive decline after diagnosis. While none of the SNPs were associated with degree of pathology, two were associated with accelerated cognitive decline and two with slower decline [43].

While the glymphatic hypothesis engendered much interest, alternative hypotheses for fluid movement within the brain were proposed. Smith et al. [49, 50] recently questioned the experimental underpinning of the glymphatic hypothesis and provided evidence that solute movement through the brain is by diffusion rather than convection. In addition, Hannocks et al. and Pizzo et al. [51, 52] provided evidence that a perivascular space is present in all vessel calibers and that fluid/solute flow may occur through that space from arteriole to capillary to venule.

Although great progress was made in imaging of the perivascular pathways, there is a need for methods to quantify the importance of different pathways within the brain parenchyma. Currently, importance is attached to experiments manipulating aquaporin-4. There are questions, however, over the impact of such manipulations on not only movement of fluid through astrocytes, but also on extracellular diffusion (e.g. volume/tortuosity of the extracellular space).

## CSF analysis

CSF analysis to aid in disease identification, progression and prognosis as well as for elucidating therapeutic targets continued to be a major focus across a wide range of neurological conditions (e.g. [53–59]). One area receiving especial attention was mild cognitive impairment and transition to dementia, particularly in relation to Aβ42 and tau. Some of the practical and theoretical difficulties in using such markers were outlined in recent reviews [55, 58, 60]. Aβ42, total tau and phosphorylated tau were also examined in relation to idiopathic normal pressure hydrocephalus [53]. One issue in CSF analysis is determining the underlying causes of altered CSF protein concentrations: changes may be due to altered barrier function or altered drainage, or both. However, a recent study found that increased CSF proteins are most probably derived from barrier dysfunction [61]. In this regard, it may be possible to determine the source of proteins based on their glycosylation state [62]. This is important because concentrations of CSF components in diagnostic studies need to be normalized to total protein content.

Studies using CSF for diagnosis are ongoing: for example, cytokines were measured in multiple sclerosis and polyneuropathy [63]. Analysis of CSF Aβ42, t-tau and p-tau may be used to distinguish Alzheimer’s disease from normal pressure hydrocephalus [53] and positive MRZ-1 antibody in CSF is a good indicator for multiple sclerosis [64]. Also, CSF chemo- and cytokines were measured in infants with post-hemorrhagic hydrocephalus [54]. There was also growing interest in disease-related changes in microRNAs, which may be encapsulated within CSF exosomes [65–67]. As well as being markers of disease processes, microRNAs may be important in cellular communication.

## Hydrocephalus

Genetic causes for congenital hydrocephalus involving abnormal brain development continue to be reported: for example loss or mutations in the MPDZ gene affected ependymal cells and led to hydrocephalus [68, 69]. Also, mice lacking the Dusp16 gene developed hydrocephalus with brain overgrowth [70] and a mutation in B3GALNT2 gene led to hydrocephalus in Mexican horses [71].

Post-hemorrhagic hydrocephalus is a major problem in infants that survive at even earlier stages of prematurity. A post mortem study of human infants found that there was extensive disruption of the ventricular zone with loss of ependyma and infiltration of astrocytes [72], consistent with a common finding in models of congenital hydrocephalus of abnormal cell junction pathology and abnormal neurogenesis (reviewed by Rodriguez and Guerra [73]). An interesting insight into post-hemorrhagic hydrocephalus was provided by Karimy et al. [26]. They found an inflammation-mediated hypersecretion of CSF after intraventricular hemorrhage in rats. This was mediated by Toll-like receptor-4 activation at the choroid plexus and resulted in activation of Ste20-type stress kinase that phosphorylated and stimulated Na–K–Cl cotransporter-1 at the choroid plexus, thus, increasing CSF secretion. Such hypersecretion may help clear blood-derived neurotoxic compounds (e.g. hemoglobin and iron) from the CSF but also may participate in generating hydrocephalus, if flow pathways are impeded.

Idiopathic normal pressure hydrocephalus (NPH) continues to generate much interest mainly because of a large increased incidence in the elderly population and the variable response to shunt surgery. A meta-analysis of published papers on CSF biomarkers concluded that CSF Aβ42, t-tau and p-tau were increased compared to the normal state [53] and that Aβ42, tau and p-tau, neurofilament light chain and leucine-rich alpha-2-glycoprotein have the greatest predictive value for improvement with shunt surgery [74]. Evolving magnetic resonance techniques showed that in NPH the CSF pulsatility was increased in the aqueduct [75], that the brain parenchyma became stiffer [76], that cerebral blood flow in selected regions including the periventricular white matter was reduced and correlated with decline in cognitive function [77]. It was found that white matter perfusion increased after shunt surgery [78], an observation consistent with improvement in fractional anisotropy of white matter tracts after shunt surgery [79].

## The meninges and other barriers

Historically, most studies on ‘barrier’ tissues focused on the cerebral endothelium or the choroid plexus. Relatively few studies examined the meninges, another site of the blood-CSF barrier. There were, however, a number of interesting studies this year that focused on the meninges and novel functions. Thus, recent evidence indicated that the meninges of perinatal mice contain neurogenic progenitor cells (radial glia-like) that can migrate into the cerebral cortex and form functional neurons [80]. In addition, Suter et al. [81] found that meninges from spinal cord produced both attractive and repulsive factors that help guide different types of axons and may regulate which axons traverse the boundary between the central and peripheral nervous systems.

Similar to the meninges, few studies have focused on glial barrier functions. Interestingly, Horng et al. [82] recently showed that reactive astrocytes around inflammatory lesions express claudin-1 and -4 and junctional adhesion molecule-A. Importantly, they found mice with astrocyte-specific knockout of claudin-4 had greater leukocyte infiltration and worse outcome in models of neuroinflammation.

## Technological advances

Many advances in brain barriers and brain fluids research are driven by technological progress. Thus, for example, efforts to improve in vitro NVU/BBB models continue. These included increased use of induced pluripotent stem cells (iPSCs) to create models of the human neurovasculature (endothelial cells alone or in co-culture with derived pericytes, astrocytes and neurons) [83–85]. In addition to expression of many classic brain endothelial markers, such models exhibited very high transendothelial electrical resistances. Recently, in vitro models were derived from iPSCs from single patients [83, 86], a system with great potential for examining the impact of patient genetics on barrier properties. The production of such cells has required a lengthy protocol, but efforts to reduce that time were reported [87]. A major area that still remains to be resolved is how well these models replicate transport at the in vivo brain endothelial cell, e.g. efflux transporter activity. Efforts also progressed using microfluidics to produce a ‘BBB-on-a-chip’ [12, 88]. Such models were extended into disease-relevant models [89].

Much new insight into barrier function and brain fluid dynamics in health and disease were derived from advances in imaging. Advances in in vivo optical imaging was the subject of a Society for Neuroscience mini-symposium [90]. In vivo imaging can be facilitated by the choice of animal models. This was exemplified by an elegant study on angiogenesis and barrier-genesis in zebrafish [91]. Advances in the use of clinical and preclinical imaging for examining the NVU was also the subject of another conference [92]. As mentioned earlier, improved techniques and better resolution in magnetic resonance images have great potential for understanding the pathology of neurological diseases with, for example, observation of disturbances in white matter tracts [79] and tracking of transependymal and periarterial flow with the aid of a contrast enhancement agent [44].

There have been initial studies involving extensive genomics and large-scale proteomics that focused on the NVU, the cerebral endothelium and the choroid plexus in health and disease [93–97]. Advances in metabolomics have yet to be extensively applied and may provide important information. In an interesting alternative approach to using liquid chromatography coupled to tandem mass spectrometry (LC–MS) based proteomics, Lee et al. [98] used the publically-accessible Human Protein Atlas (mostly immunohistochemistry based) to examine the human cerebrovascular distribution of 20,000+ proteins. An affiliated database allowed comparisons between cell types within the brain and across organs. It also provided information on endothelial heterogeneity (e.g. by vessel caliber or adjacent cells), an understudied area.

## Future directions

Major progress is being made in our basic science of the brain barriers and brain fluids, although there are major controversies. The ultimate goal of such understanding is, however, translating that information to the clinic. While there are some clinical trials, history shows us the difficulties in such translation.
